# The Reliability of a Functional Agility Test for Water Polo

**DOI:** 10.2478/hukin-2014-0046

**Published:** 2014-07-08

**Authors:** Guilherme Tucher, Flávio Antônio de Souza Castro, Nuno Domingos Garrido, António José Rocha Martins da Silva

**Affiliations:** 1 University of Trás-os-Montes and Alto Douro, Vila Real, Portugal.; 2 Federal University of Rio Grande do Sul, Porto Alegre, Rio Grande do Sul, Brazil.

**Keywords:** Water polo, agility assessment, reproducibility, testing

## Abstract

Few functional agility tests for water polo take into consideration its specific characteristics. The preliminary objective of this study was to evaluate the reliability of an agility test for water polo players. Fifteen players (16.3 ± 1.8 years old) with a minimum of two years of competitive experience were evaluated. A Functional Test for Agility Performance (FTAP) was designed to represent the context of this sport. Several trials were performed to familiarize the athlete with the movement. Two experienced coaches measured three repetitions of the FTAP. Descriptive statistics, repeated measures analysis of variance (ANOVA), 95% limit of agreement (LOA), intraclass correlation coefficient (ICC) and standard error of measurements (SEM) were used for data analysis. It was considered that certain criteria of reliability measures were met. There was no significant difference between the repetitions, which may be explained by an effect of the evaluator, the ability of the players or fatigue (p > 0.05). The ICC average from evaluators was high (0.88). The SEM varied between 0.13 s and 0.49 s. The CV average considering each individual was near 6–7%. These values depended on the condition of measurement. As the FTAP contains some characteristics that create a degree of unpredictability, the same athlete may reach different performance results, increasing variability. An adjustment in the sample, familiarization and careful selection of subjects help to improve this situation and enhance the reliability of the indicators.

## Introduction

Water polo is a sport that involves acyclic movements and complex motor coordination (Kos et al., 2010; Lozovina et al., 2010). Movements that are close to the goal and independent of the player’s position are activities of greater intensity, such as blocking, ball disputes, direct contact with the opponent and explosive actions that normally last between 1.5 and 6.3 s (Tan et al., 2009). In spite of the importance of movements near the goal during actions of attack and defense, the majority of functional agility studies focus on the physical fitness of the player when displacing horizontally (Rechichi et al., 2000; Rechichi et al., 2005; Mujika et al., 2006; Tan et al., 2010). These quick horizontal displacements are more frequent in counter-attack activities, especially when there is a large difference in the team’s performance. However, when the two teams are technically and tactically well-matched, the greatest activity occurs close to the goal (Lupo et al., 2010; Lupo et al., 2011).

When the athletes are positioned close to the goal they need to change their body from a horizontal to a vertical position, in various directions and different planes and therefore require agility. The ball is disputed with the opponent and a set of rapid rotational movements and positions are taken with the objective to execute determined defensive or offensive tactics (Smith, 1998; Tan et al., 2009; Lupo et al., 2010; Lupo et al., 2011). These moderate to high intensity agile actions can occupy up to 50% of the game time (Smith, 1998). However, currently there is no functional agility test that assesses these movements in water polo. Thus, it is necessary to construct a test to evaluate the specific agility of players that, when combined with their physical motor abilities (Sheppard and Young, 2006; Sheppard et al., 2006; Currell and Jeukendrup, 2008; Veale et al., 2010; Young and Willey, 2010) represents the specific requirements of the sport (Tan et al., 2009).

Sheppard and Young (2006) and Young et al. (2002) define agility as the capacity of an athlete to rapidly change speed or direction in response to a stimulus. Agility is an important quality in team sport games and quick decision-making is an important factor in agility performance. Therefore, the player’s perception abilities can influence performance and should be considered during the assessment (Sheppard and Young, 2006; Young and Willey, 2010). Agility tests normally evaluate the capacity of the athlete to move quickly in one direction. However, recent studies have highlighted the importance of an evaluation of a combined set of cognitive and physical components (Sheppard and Young, 2006). As such, besides the athlete having to quickly change direction, this action would have to be in response to an unexpected situation occurring during the test (Sheppard and Young, 2006; Veale et al., 2010; Young and Willey, 2010).

Optimum performance depends on strength, power, technique, cognitive capacity, overall vision of the surroundings, alertness and anticipation (Young et al., 2002). The measurement of performance is one of the most important measures in sport science. However, some factors can influence these measurements so that they do not represent the athlete’s true performance (Atkinson and Nevill, 1998; Bland and Altman, 1999). Consequently, measurement reliability is a top priority when designing and executing a test (Atkinson and Nevill, 1998). Reliability refers to consistency in the reproduction of the measurements. This indicates that the test is able to reproduce similar measurements under different circumstances or that measurements can still be evaluated when there is a deliberate absence of an intervention that generates a change (Currell and Jeukendrup, 2008).

Taking into consideration the above requirements, the objective of this paper was to present the results of a preliminary study aimed to test the reliability of a functional test for agility performance (FTAP) for youth water polo players.

## Material and Methods

### Participants

Fifteen youth, male competitive water polo players with a minimum of 2 years experience and with different ability levels were evaluated. Their ability levels varied in accordance with their expertise and tactical position. The average age of the athletes was 16.3 ± 1.8 years. The project for this study was approved by the Ethic Committee under the number of 70263/2012 and in accordance with the Declaration of Helsinki.

### Measures

**Figure 1:**
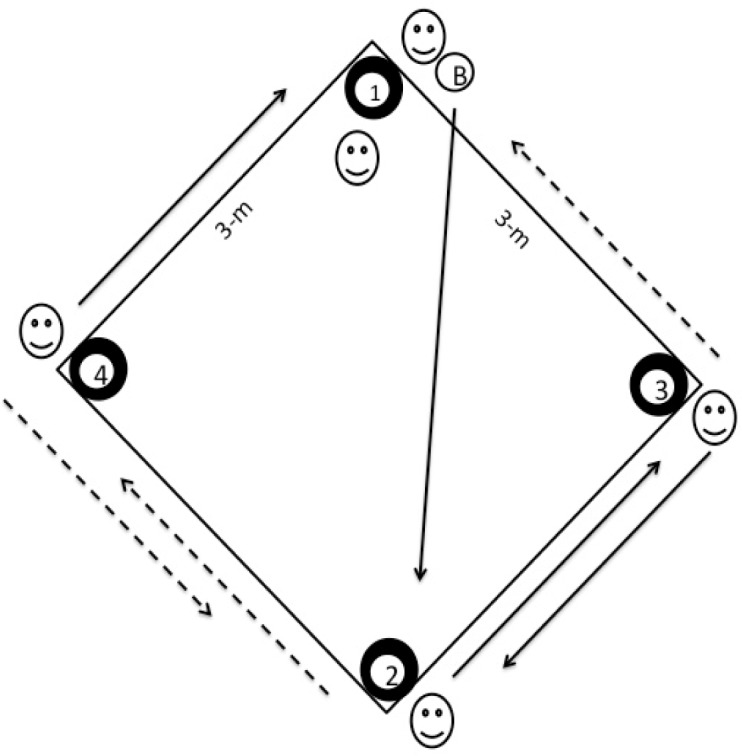
Schematic representation of the Functional Test for Agility Performance (FTAP) proposed to evaluate water polo players

To evaluate the specific agility of the players the authors designed a Functional Test for Agility Performance (FTAP). The test is characterized by its open nature (Sheppard and Young, 2006; Veale et al., 2010; Young and Willey, 2010), since the athlete does not have prior knowledge of the direction of his displacement thus ensuring the randomness of movements generated by the passes made by another player. The test presents the subjects with high-intensity dislocation of short duration, similar to the situations indicated by Tan et al. (2009). The FTAP’s scheme is presented in Figure 1.

The evaluations were performed in a 3-meter-sided square area marked with PVC pipes of 0.02 m thickness. Adequate buoyancy was guaranteed by the fixation of the PVC pipes to floating material. At each corner of the square flexible floating arches of 0.80 m were placed, attached by a pipe; they were responsible for keeping the ball in the desired area. The arches were fixed to the PVC pipes with a hook-and-loop fastener. The evaluation area was kept in place with the use of a cord tied to the pool’s lane markers. The distance between the evaluation area and the person evaluating the athlete did not exceed 5 m.

### Procedures

The athletes were instructed as a group regarding the test procedure. Following this, two FTAP familiarization repetitions were permitted. This was the same for both the athlete being tested and the other players responsible for the passes. Doubts or queries raised by the subjects were addressed, ensuring that all subjects understood the procedure before actual testing.

The FTAP testing occurred between 3:00 and 5:00 p.m.. The participants had been advised not to exercise in the morning and two hours before testing time. A midday meal was to be eaten at least 90 minutes before the commencement of the test. There was a standardized warm-up consisting of dry-land stretching and dynamic articular mobility exercises including 200 m free style swim alternating front and back strokes and various kick styles, 4 × 100 m front crawl swims with no-push turns every 25 m, starting every 110 seconds and 4 × 25 m (12.5 m sprint, 12.5 m recovery), starting every 50 s. Due to the testing conditions, evaluations were conducted individually for each subject and the warm-ups organized in such a way as to ensure that the test was performed no more than 5 minutes following the warm-up.

The player being tested was within the FTAP square, at one of its extremities and had one hand on a ball that was floating in the arch near him. This was considered the start (Picture 1a). Another four players were positioned outside each of the four FTAP square corners with one ball in each arch (Picture 1a and Figure 1; in Figure 1, numbers 1–4 represent the 4 balls and their respective arch and athletes). The player next to the subject being tested (arch 1) had a fifth ball in his hand (Figure 1; circle with letter B; Picture 1a). When this player perceived that the tested player had removed his hand from the ball, thus beginning a fast displacement to the center of the square, he threw the ball immediately to the player at the opposite extremity (Picture 1b and Figure 1; in Figure 1, the player in arch 1 passed the ball to the player at arch 2). Upon receiving the ball, this player (arch 2) then passed the ball to one of the players at his side (arch 2 player can pass the ball to a player at either arch 3 or 4; Picture 1c; the player in arch 2 passed the ball to the player at arch 4) without indicating the intended direction or exercising movements that may trick the tested player. When this movement occurred, the player being tested should move as quickly as possible to where the ball had been passed and remove a ball that was floating in the arch using any part of his body (Picture 1c and 1d). The player who received the ball (arch 3 or 4) should then pass it once again (the player at arch 3 or 4 can only pass the ball to the players at arch 1 or 2, not to each other; Picture 1d; the player in arch 4 passed the ball to the player at arch 1). The test was then completed.

It is important to note that the tested player does not know in advance to whom the ball will be passed. In addition, the four other players and the destination of the passes are randomly chosen, being different for each of the repetitions. The tests were repeated three times for each individual from a randomly determined list according to that proposed by Hopkins (2000). For this test, a three-minute rest interval was allowed between repetitions. If any factor occurred that may have hindered the performance of a normal test (tested player’s displacement error or wrong pass, for example), the procedure for the same tested player was performed after the next athlete in line was tested.

Time was manually measured in seconds using two sport chronometers (Professional Stopwatch with USB – model JS-9006P) by two experienced water polo coaches, named evaluator A and B. The evaluators were informed of the FTAP procedures and together with the athletes, were familiarized with the test. The evaluator began timing the test from the moment the tested player removed his hand from the ball in arch 1. Timing stopped when the tested player removed the second ball from the arch (Picture 1d), giving the total time for the test. To avoid interference in the test performance, the athletes did not receive any information about the time results until the end of the test.

### Statistical Analysis

The descriptive statistics included the average values, standard deviation (SD), and coefficient of variance. The normality of all the measurements was achieved using the Shapiro Wilk test. An ANOVA for repeated movements in a mixed 3×2 model (repetition × evaluator) was used to test the influence of factors (evaluator, repetition and interaction between evaluator and repetition) on the results. The Mauchly test was used to test the sphericity assumption for the evaluator effect, the repetition and the evaluator × repetition interaction. In all cases, an alpha < 0.05 was considered statistically significant.

Measurement variation quantification from evaluators A and B followed that previously proposed by Bland and Altman (1999). The average measurements between the evaluators for the three repetitions were then considered (Bland and Altman, 1999). The 95% limit of agreement (LOA) was calculated by summing up the difference between the averages from evaluators A and B (d) with a product of ± 1.96 multiplied by the SD of the difference between the averages of evaluators A and B (SD) (thus, LOA = d ± 1.96^*^SD).

The procedures used to calculate the intraclass correlation coefficient (ICC) took into consideration the studies of Shrout and Fleiss (1979), McGraw and Wong (1996) and Weir (2005). A two-way random model of the absolute confidence type was utilized. ICC was calculated between each of the repetitions registered by the evaluators A and B (1^st^ vs. 1^st^; 2^nd^ vs 2^nd^; 3^rd^ vs. 3^rd^). Simultaneously, calculations were performed for only those repetitions reported by evaluator A; then only for those from evaluator B; and finally for the average of the values from evaluator A and B. It had previously been advised that the ICC should be greater than 0.9, however, such reports also indicate that the ICC value should be interpreted in accordance with the nature of the designed test (Atkinson and Nevill, 1998).

The standard error of measurements (SEM) represents a variation among individuals and is expressed by the square root of the average quadratic error of two-way ANOVA for repeated measurements (Eliasziw et al., 1994; Atkinson and Nevill, 1998). The smaller the SEM, the greater the reliability of the measurement (Atkinson and Nevill, 1998). The statistics were treated by IBMSPSS version 20 software.

## Results

The average coefficient variation (CV) of the measurements, considering each individual, from evaluator A was 6.97%, and from evaluator B, 6.20%. Considering each repetition, this value was 9.25% from both evaluators. The CV for each measurement from the evaluators is shown in Table 1, together with data from the descriptive statistics.

There were no significant differences between the repetitions that could be explained by the effects of the evaluator, the ability of the players or fatigue. As such, the results for the evaluators (F_1, 14_ = 1.41; p = 0.25), the repetitions (F_2, 28_ = 0.47; p = 0.63) and the interaction between the evaluator and repetition (F_2, 28_ = 1.13; p = 0.33) indicate that the time measured by the evaluators had no influence on the results, regardless of the repetition performed.

**Figure 2:**
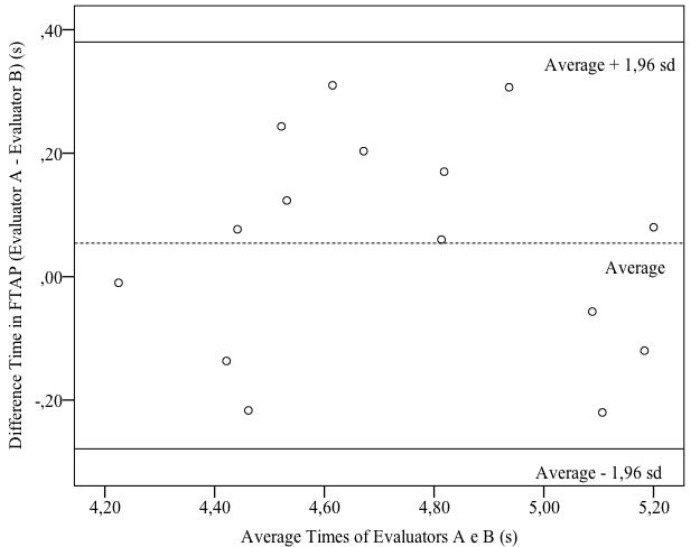
FTAP performance time: difference of time (evaluator A minus evaluator B) versus average time measured by evaluators A and B with the 95% limit of agreement (sd = standard deviation)

The average difference (evaluator A minus evaluator B) was 0.054 s and the SD was a difference of 0.17 s. The difference presented a normal distribution (p = 0.50). Therefore, it could be expected that in 95% of the cases, the difference between the measurements registered by the evaluators would be between − 0.28 s (average – 1.9650) and 0.38 s (average + 1.9650), which characterizes the 95% limit of agreement (LOA). These values represent an amplitude for the value obtained of 0.66 s (Bland and Altman, 1999) (Figure 2).

The intraclass correlation coefficient (ICC) for the analyzed moments is shown in Table 2. The best ICC values were found between the 1^st^ and the 3^rd^ repetitions registered by evaluators A and B (ICC = 0.87). The same can be said about the average measurements obtained from the evaluators (ICC = 0.88).

The standard error of measurements (SEM) varied between 0.13 s and 0.49 s, depending on the considered situation. The complete results are shown in Table 3.

## Discussion

The primary objective of the present study was to test the reliability of a Functional Test for Agility Performance (FTAP) used to evaluate youth water polo players. In relation to the experimental design used for this study, the results indicate that whilst this test requires further adjustments to some parameters, it meets the necessary criteria indicated in the literature (Eliasziw et al., 1994; Atkinson and Nevill, 1998; Bland and Altman, 1999; Hopkins, 2000; Bland and Altman, 2003; Weir, 2005). This is the first study addressing the reliability of a FTAP for water polo. Whilst it is not possible to compare the performance of these athletes with others, it is however believed that more experienced players could most likely complete the test in fewer seconds. In the same way, the individual CV could also be lower in more experienced players.

This study was performed with 15 youth water polo players, each with ability levels in accordance with their expertise and tactical position. Reliability studies for water polo (Mujika et al., 2006; Platanou, 2006; Tan et al., 2010) have used a number of players less or near to the sample size of this study. For this type of research, however, it is recommended that a sample size of around 50 individuals should be used (Hopkins, 2000). Together with the relatively small sample size, there was an attempt to present in the FTAP the condition of uncertainty that naturally occurs in a competitive game, which has yet to be reported in the literature. These characteristics of uncertainty involved, as observed in the results, an increased chance for variation in the results of test-retest values but not in the time measured by different evaluators of the same repetition. Therefore, these two factors may hinder the homogeneity of the group’s performance, which is not recommended (Alricsson et al., 2001). Whilst there was no significant difference in the measurements, these uncertainties none-the-less resulted in an increase in variation in the results.

The average CV for the measurements for each individual taken by evaluators A and B was around 6%. According to existing literature, an acceptable value is under 10% (Atkinson and Nevill, 1998). Individually, some of the athletes in the current study presented a high CV (> 8%), thus influencing the average CV. As previously mentioned, this is one of the characteristics that cannot be predicted and one that affects the performance of athletes with lesser capability. In practical terms, a CV of 6% for an average time of 4.70 s represents a variation of 0.28 s and the variation observed in the current study was similar to those reported by Alricsson et al. (2001) although the time for that test was approximately 10 s. Another difference was that Alricsson et al. (2001) evaluated velocity and agility in closed tests.

The SEM and the ICC values present a different interpretation in accordance with the situation in which they were calculated. The most generic understanding of these results however, indicate that in all moments that consider the effect of repetition, the SEM and ICC were the worst. A comparison between the measures obtained by the evaluators for each repetition separately presented an adequate ICC (with the exception of the second repetition which presented a lower ICC). The same occurred for the SEM, with a difference between the evaluators of less than 0.20 s. However, when the repetition effect considered to obtain these measurements was less than expected, the SEM was approximately 0.40 s. Once more, the influence of non-systematic variation was noticeable, as represented by the repetition effect (variability of the results among repetitions).

The Bland-Altman graphic analysis provides a better vision of the agreement between the evaluators to obtain the measurements (Bland and Altman, 1999). When the time measured by the two evaluators is compared, it is expected that the average difference between the two measurements will be zero, indicating the absence of a difference (Bland and Altman, 1999). However, this is a technical concept and in truth, the least possible difference is expected. In the present study, this value was around 0.05 s and the maximum difference predicted between the measurements from the two evaluators for the same individual, a little more than 0.5 s (LOA: – 0.28 to + 0.38). The quality of these values depends on a careful interpretation of the results and what is being treated (Bland and Altman, 1999; Bland and Altman, 2003). Therefore, for the proposed FTAP, it was considered acceptable that there would be a maximum difference of around 0.5 s between the two evaluators in the evaluation of the same individual. It is worth mentioning that in the majority of cases, in water polo teams, there is only one evaluator present who is responsible for this task.

There was no significant effect of the action of the evaluators on the measurement of the repetitions or the interaction between the two evaluators for the times obtained in the FTAP. This indicates that there was no effect (including fatigue) that modified or affected the measurements obtained by the two evaluators during the repetitions. It must be noted however that the value of the F ratio is a variable explained by the ratio between systematic and non-systematic variations. The systematic variation is explained by the model and takes into consideration the influence of the experimental effect. The non-systematic variation indicates the influence of extraneous factors. The greater the influence of the non-systematic variation (compared to the systematic variation and represented by an F value below one), the less chance of finding a significant difference between the measurements. Thus, the repetition effect in the present study (F = 0.47) indicates a greater non-systematic variation influence on this factor (Hopkins, 2000; Field, 2009).

In the present study, the following criteria were considered non-systematic variation effects: (1) the attention of the evaluator in measuring the time; (2) understanding of the test of all involved subjects; (3) the sports ability of the tested player; (4) the influence of the athlete who passed the ball and the response of the tested player to it; (5) the correct utilization of the arm that removed the ball from the first floating arch, facilitating the following movements; and (6) the natural improbability of the test, as it has the characteristics of being an open test – similar to that of a real game, whereby the athlete’s behavior is unpredictable. For optimal reliability, however, repetition is of extreme importance as it generates similar measurements in the test-retesting of the athlete. A way in which to ensure this similarity is to guarantee that every participant is familiar with the necessary number of repetitions required to achieve this. Even though the test in the current study was thoroughly and suitably explained and more familiarization repetitions were performed (2 vs. 1) when compared with the experimental design applied in previous studies (Rechichi et al., 2000; Mujika et al., 2006; Platanou, 2006; Tan et al., 2010), it is believed that due to the nature of the variability and unforeseeable behavior during the FTAP, this number could be higher.

Studies such as that of Moir et al. (2004) ruled out the need to familiarize tested subjects as the participants would then know in advance the actions that would be executed. However, these actions are far from the reality encountered in competitive games and serve more to evaluate the physical performance of the player (Sheppard and Young, 2006; Sheppard et al., 2006; Currell and Jeukendrup, 2008). Team games are characterized by their complexity, and consequently by their difficulty to measure the player’s performance (Currell and Jeukendrup, 2008).

As a result of the previously explained factors, it is believed that an increase in the number of familiarizing repetitions was a limitation of this study in that it may have diminished the performance variability of the individual in the test-retest and improved the competency of those participating in the FTAP procedure (the athlete being tested, the evaluator, and the athletes performing the passes). The fact that a manual chronometer was used for the FTAP measurements could also have some effect on the test results. However, this procedure was conducted by two evaluators for all repetitions and no significant difference was found. Hence, it is regarded that because the greater variation was found between the repetitions and not between the evaluators, the evaluators were sufficiently familiar with the test procedures and concentrated their efforts on obtaining precise measurements for the repetitions.

Similar to the present study, Alricsson et al. (2001) used a manual chronometer for marking time and admitted that this variable could have possibly affected the measurements, even though the reliability criteria had been satisfied. This indicates that there is a need to stabilize the measurements with the aid of electronic timers. At the same time however, it is believed that as this test is performed in water, the aforementioned initiative would make the FTAP procedure too expensive and would consequently not be frequently implemented by competitive teams. The challenge, then, is to find adjustments that match the reliability criteria but maintain the simplicity and practicability of the test. As pointed out by Atkinson (2002), athletes benefit from measured values and not from hypothetical notions. It is vital to determine the mechanisms that caused an undesirable effect during the measurement so that the obstacles can be overcome.

It can be concluded that the Functional Test for Agility Performance (FTAP) for young water polo players reported in the current study, presented good reliability between the evaluators for the criteria under consideration within the experimental design for the test-retest procedure. It is believed that some adjustments regarding sample size, performance homogeneity of the athletes, and improved familiarization of the test procedures by those involved are required. These adjustments would ensure less variation in the performance measurements of the repetitions, and consequently improvement of the reliability indexes.

## Figures and Tables

**Picture 1:**
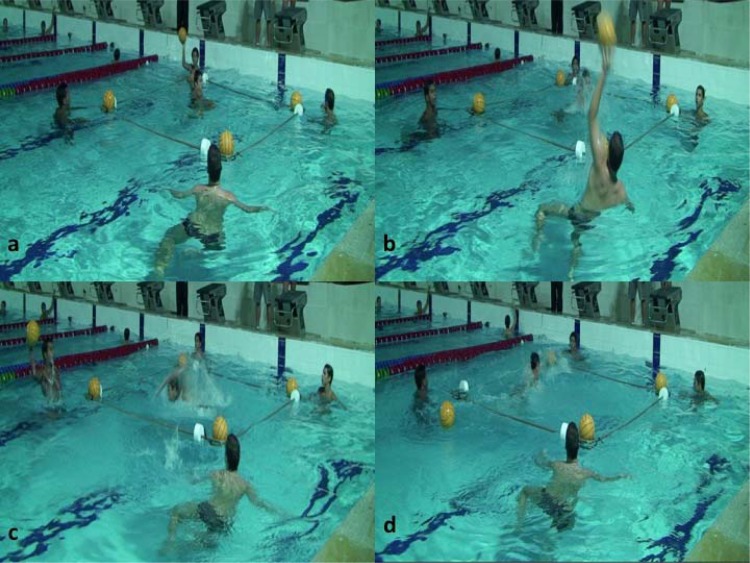
Functional Test for Agility Performance (FTAP) to evaluate water polo players Picture 1a. Start of the test - the player being tested is within the FTAP square and has one hand on a ball. Picture 1b. First pass – tested player moves to the center of square. Picture 1c. Second pass - tested player moves where the ball has been passed and removes a ball that is floating in the arch. Picture 1d. Third pass – tested player moves where the ball has been passed again and removes a ball that is floating in the arch. The test is then completed.

**Table 1:** Results of the descriptive measurements of the repetitions during FTAP as registered by evaluators A and B for each repetition

Measure	Evaluator A				Evaluator B			

	1^st^	2^nd^	3^rd^	Total	1^st^	2^nd^	3^rd^	Total
Average	4.73	4.84	4.72	4.76	4.75	4.75	4.62	4.71
sd	0.51	0.44	0.37	0.44	0.55	0.35	0.41	0.44
S^2^	0.26	0.20	0.14	0.20	0.30	0.12	0.17	0.20
CV	10.72	9.13	7.88	9.25	11.53	7.38	8.84	9.25

Average, standard deviation (sd), variance (S^2^) and coefficient of variation (CV)

**Table 2:** ICC results for different FTAP conditions

Conditions	ICC	95% IC
1^st^ A and B repetition	0.87	0.65–0.95
2^nd^ A and B repetition	0.67	0.27–0.87
3^rd^ A and B repetition	0.87	0.61–0.95
Evaluator A repetitions	0.26	-
Evaluator B repetitions	0.40	-
All A and B repetitions	0.40	0.19–0.66
Average A and B repetitions	0.85	0.61–0.94

**Table 3:** Standard error of measurements (SEM) results under different FTAP conditions

Conditions	SEM (s)
Between 1^st^ A and B repetitions	0.19
Between 2^nd^ A and B repetitions	0.23
Between 3^rd^ A and B repetitions	0.13
Between measures of A	0.38
Between measures of B	0.35
Between measures considering evaluators effect	0.21
Between measures considering repetitions effect	0.49
Between measures considering evaluators and repetitions effects	0.17
